# Serum anti-Müllerian hormone as a predictor of polycystic ovarian syndrome among women of reproductive age

**DOI:** 10.1186/s12905-022-01782-2

**Published:** 2022-05-28

**Authors:** Muhammad Salman Butt, Javeria Saleem, Sobia Aiman, Rubeena Zakar, Iftikhar Sadique, Florian Fischer

**Affiliations:** 1 Department of Public Health, University of the Punjab, Lahore, Pakistan; 2 Akhtar Saeed Medical and Dental College, Lahore, Pakistan; 3 Institute of Public Health, Charité – Universitätsmedizin Berlin, Charitéplatz 1, 10117 Berlin, Germany

**Keywords:** Anti-Müllerian hormone, PCOS, Rotterdam criteria, Hirsutism

## Abstract

**Background:**

Polycystic ovarian syndrome (PCOS) affects up to one-fifth of women of reproductive age and causes anovulatory subfertility. Some studies have recommended that an anti-Müllerian hormone (AMH) level greater than 3.8–5 ng/mL can be used for diagnosing PCOS. This study aims to analyse serum AMH levels among PCOS women of reproductive age to use AMH as a biomarker predictor along with other Rotterdam criteria.

**Methods:**

In this cross-sectional study, a total of 98 women visiting the fertility center of a private hospital in Lahore, Pakistan, were screened. Data were obtained from 51 PCOS newly diagnosed women aged 28.24 years (SD ± 4.84 years) meeting at least two of the Rotterdam criteria and specific inclusion criteria. Baseline variables, menstrual cycle length, ovarian morphology on ultrasound, hirsutism, sex hormones, gonadotropin, and serum AMH levels were analysed during the follicular phase (1–5 days) of the menstrual cycle. Serum AMH was measured by an enzyme-linked immunosorbent assay.

**Results:**

A high serum AMH level (7.23 ± 4.67 ng/ml) was recorded with normal sex hormone levels. Women with oligo-/amenorrhea had a significant mean difference for luteinizing hormone (p = 0.02) and AMH levels (p = 0.03) when compared with women of normal menstrual cycle length. PCOS women with high AMH levels (≥ 3.9 ng/ml) showed a significant difference in ovarian morphology (p < 0.05) when compared with the normal AMH group.

**Conclusions:**

An elevated serum AMH level can be used as a strong predictor to reflect the certainty of PCOS diagnosis among women of reproductive age when study concurrently with the other Rotterdam criteria.

## Background

Polycystic ovarian syndrome (PCOS) is one of the most prevalent endocrine abnormalities for women of reproductive age. The prevalence of PCOS varies among ethnic populations and appears to decrease with age. It is estimated that British women aged 20–25 years have a prevalence of 33% [1]; Finnish women aged less than 36 years have a prevalence of 21.6% [2], and the prevalence is 21% in New Zealand [3] and 23% in Australia [4] among reproductive-aged women. The prevalence of PCOS in South Asian women, especially in Pakistani women, is much higher (52%) than that in the white population (20–25% in the UK) [5].

PCOS was initially described by Stein and Leventhal in 1935, although it had a history of at least a century beforehand [6]. Stein and Leventhal initially defined this disorder as enlarged ovaries, hirsutism, obesity, and anovulation. Rotterdam criteria described PCOS as a disorder with at least two of the clinical features: (1) oligo- or anovulation, (2) clinical or biochemical signs of hyperandrogenism, and (3) polycystic ovaries, with the exclusion of other aetiologies (congenital adrenal hyperplasia, androgen, secreting tumours, Cushing’s syndrome) [7].

Women with PCOS can be classified into four potential phenotypes based on history, physical examination, and investigations [8]: (1) PCOS complete, which is oligo-/anovulation (O) + polycystic ovaries (P) + hyperandrogenism (H), (2) polycystic ovaries + oligo-/anovulation, (3) polycystic ovaries + hyperandrogenism, and (4) oligo-/anovulation + hyperandrogenism [9]. A transvaginal ultrasonogram is commonly used to measure antral follicles, size, and ovarian volume. An ovarian volume greater than 10 mL and the occurrence of 12 follicles of 2 to 9 mm represents the typical polycystic ovarian morphology (PCOM) as per the Rotterdam diagnostic criteria [7, 10].

The clinical manifestations of insulin resistance, hyperandrogenism, and sarcopenic obesity are common among PCOS women. Peripheral insulin resistance results in beta cell dysfunction, causing PCOS women to have type II diabetes. A prevalence of 55% for insulin resistance has been found among PCOS women regardless of their BMI status [11]. Insulin resistance and compensatory hyperinsulinemia act synergistically with luteinizing hormone to cause hyperandrogenism among women with many PCOS phenotypes [12]. This change in the pathophysiological mechanism may contribute to an increase in the risk of pregnancy-induced hypertension, gestational diabetes mellitus, spontaneous preterm birth, and maternal, fetal, and neonatal complications among PCOS pregnant women [13].

A prevalence of 55% for insulin resistance has been found among PCOS women regardless of their BMI status [11]. An insulin resistance test to diagnose PCOS is not needed; instead, obese women should initially be screened for metabolic syndrome, which should be correlated with a family history of diabetes [7]. D-Chiro-inositol (DCI) and myo-inositol (MI) showed a safe nutraceutical profile and helped to improve insulin resistance and related metabolic symptoms among PCOS women. A recommended dose of 4 g MI/day has been previously reported to also improve the regulating activities of several reproductive hormones, including follicle-stimulating hormone (FSH) and thyroid-stimulating hormone (TSH) [12].

Establishing a diagnosis of PCOS is difficult among adolescents and menopausal women [14]. The features of PCOS begin at menarche but appear after puberty. Several hormonal factors are responsible for promoting such risk factors, including high levels of gonadotropin-releasing hormone (GnRH), follicle-stimulating hormone (FSH), luteinizing hormone (LH), insulin, androgen, anti-Müllerian hormone (AMH), vitamin D deficiency, and calcitonin. The LH level is often two to three times that of the FSH level. It is typical for women with PCOS to have an LH level of approximately 18 IU/mL and an FSH of 6 IU/mL. LH hypersecretion, both basally and in response to GnRH, is a characteristic hallmark of PCOS and can be recognized as the primary abnormality in classic PCOS causing androgen excess [15].

Small antral (< 2 mm) and preantral ovarian follicles produce AMH, which is an essential primitive factor for folliculogenesis and PCOS identification markers. The serum AMH had a higher sensitivity than the antral follicle count, as it reflects the small antral and preantral ovarian follicles [16]. Studies have shown a strong association between AMH and hyperandrogenism and antral follicle reserves in the body, suggesting that AMH is a strong tool to diagnose PCOS [17].

Serum AMH levels are reported to be high among PCOS women because of simultaneous activation of multiple antral follicles and increased AMH production per follicle. There is limited evidence to define the cut-off value for AMH as a diagnostic tool for PCOS. Some studies have recommended that an AMH level greater than 3.8–5 ng/mL can be used as a diagnostic factor for PCOS [18] and suggested using Rotterdam criteria and AMH levels concurrently for early and accurate diagnosis. Approximately 60% of PCOS women reported having a high serum AMH value (median 5.62 ng/mL) and low pregnancy outcomes during controlled intrauterine insemination cycles [19]. The presence of two out of three clinical features (OA, HA, and AMH) was found to have 96% sensitivity and 100% specificity among patients previously diagnosed with PCOS according to the Rotterdam criterion [20].

The diagnosis of PCOS among the reproductive age group using AMH level has been studied in many populations, but evidence from Pakistan is missing on AMH level association with PCOS. Furthermore, unmarried women and adolescent girls in Pakistan are reluctant to opt for the transvaginal ultrasonogram method for PCOM identification because of the social and religious stigmas attached to it. The use of serum AMH levels to detect ovarian dysfunction as a biochemical marker for early PCOS identification will be of utmost importance for these women. This study aims to analyse serum AMH levels among PCOS women of reproductive age to use AMH as a biomarker predictor along with other Rotterdam criteria. This study hypothesized that a serum AMH level above 3.9 ng/mL can be used to identify ovarian dysfunction among PCOS women as an auxiliary investigation to transvaginal ultrasonogram. This study will also help to understand the pattern and utilization of serum AMH levels as a diagnostic factor along with other Rotterdam criteria among PCOS women.

## Materials and methods

### Study design and setting

A cross-sectional study design was used to measure the reproductive characteristics and hormonal levels among PCOS women. This study is a part of the doctoral thesis project titled “Association of Physical Activity and Dietary Habits with Vitamin D and anti-Müllerian Hormone among Polycystic Ovarian Syndrome Women in Lahore, Pakistan” by the first author (MSB). Identifying PCOS using serum AMH levels is one of the objectives of this Ph.D. Project. Data were collected from June 2019 to April 2020 from the outdoor fertility center of a private hospital in Lahore, Pakistan. Consultant gynecologists (authors SA and IS) examined the participants to be recruited for the study.

### Study population

The inclusion criteria for this study were women aged 15–45 years, with both ovaries intact, who were newly diagnosed with PCOS by consultant gynaecologists following a transvaginal pelvic ultrasound (Toshiba Xario Prime, Crawley, UK) investigation for PCOS confirmation and measuring ovary volume. According to the Rotterdam criteria, the antral follicle count on ultrasound is related to polycystic ovarian morphology (PCOM). Modern technology has improved follicle detection on ultrasonography, but it still requires specific equipment [21]. PCOM is diagnosed at the appearance of ≥ 12 follicles located on the periphery of the central stroma or at an ovarian volume > 10 cm^3^ [22].

All participants were in good health and were not taking any medication known to affect sex hormones or metabolism three months before participating in the study. Women with moderate to severe endometriosis, a previous history of surgery, and preexisting diabetes mellitus before the diagnosis of PCOS were excluded from the study after being clinically assessed by experienced physicians.

### Sampling technique

A purposive sampling technique was used to collect data. A total of 98 newly diagnosed women with PCOS were screened for participation in this study. Data were obtained from 51 PCOS-diagnosed women aged 28.24 years (SD ± 4.84 years) meeting at least two of the Rotterdam criteria and specific inclusion criteria. Women with any comorbidity and previous history of surgery were excluded from the study. The World Health Organization sample size determination in health sciences software version 2.0 was used to calculate the sample size. In Pakistan, the prevalence of PCOS was reported among 50% of women of the reproductive age group [23]. Based on this evidence, an anticipated population proportion (P) of 0.50 with an absolute precision (d) of 0.10 at a confidence level of (1-α) 95% was used to calculate the sample size. The following formula estimated a sample size of 98 for a population proportion with specified precision. Out of these screened patients, almost a 50% response rate was observed, and 51 participants were selected for data collection.

### Data collection

The consultant gynecologists clinically assessed the participants and recorded detailed menstrual cycle history and reproductive hormonal analysis. Study participants were categorized into normal and oligo-/amenorrhea groups based on their menstrual cycle history for statistical analysis. The normal menstrual cycle was defined as women with regular cycles from 22 to 30 days with an estimated average of 28 days. Women with menstrual cycle intervals ≥ 35 days (4–8 periods per year) were grouped into oligo-/amenorrhea.

Baseline information on age, marriage duration, fertility status, menstrual cycle length, and previous history of treatment was measured using a demographic questionnaire after the verbal consent of the participants. The weight of participants was measured by standing on a digital weighing scale (Beurer BF 600) with normal clothing, without shoes, and was rounded to the nearest 100 g.

Clinical signs of hirsutism were assessed by the modified Ferriman-Gallwey (MFG) scale, and a score greater than 8 was defined as hyperandrogenism [24]. Based on the MFG score, hyperandrogenism was categorized into normal (< 8), mild (8–15), moderate (16–25), and severe (> 25). The biochemical evaluation included serum follicle-stimulating hormone (FSH, mlU/ml), luteinizing hormone (LH, IU/L), prolactin (ng/ml), anti-Müllerian hormone (AMH, ng/ml), and thyroid-stimulating hormone (TSH, IU/L) during the follicular phase (1–5 days) of the menstrual cycle. Serum LH, FSH, and prolactin were measured using automated chemiluminescent immunoassays (Abbott Diagnostics, Maidenhead, UK). Serum AMH was measured by enzyme-linked immunosorbent assay (ELISA) according to the manufacturer’s instructions (DRG^®^ AMH ELISA, EIA-5738).

### Data analysis

The data were analysed using IBM SPSS version 23.0 and GraphPad Prism version 9. The demographic and reproductive characteristics are presented in absolute and relative values, as well as in mean (M) and standard deviation (SD). An independent T test was used to compare the mean differences in the reproductive hormones between the women with the normal menstrual cycle and oligo-/amenorrhea. The reproductive characteristics of the PCOS women were compared using the independent T test between the groups with normal (≤ 3.9 ng/ml) and high AMH (> 3.9 ng/ml) levels. A linear regression model was used to predict the relationship between AMH levels and reproductive characteristics of PCOS women.

### Ethical considerations

This study was approved by the University of the Punjab Research and Advanced Studies Board (ASRB) (ref # D-6067-ACAD) and the Institutional Review Board (IRB) for ethical considerations (ref # D/2022/UZ). All recruited participants provided informed consent before inclusion in the study following the guidelines of the Declaration of Helsinki [25].

## Results

A total of 51 women with a mean age of 28.24 years (SD ± 4.84) who were married for 4.62 years (SD ± 3.58) participated in this study. Mild (43.1%), moderate (35.3%), and severe hirsutism (11.8%) were recorded among the PCOS women representing the clinical signs of hyperandrogenism. Participants had menstrual cycle irregularities (39.29 ± 25.35 days) with an oligo-/amenorrhea (64.7%) pattern. Polycystic ovarian morphology (PCOM, Vol > 10 cm^3^) was evident among PCOS women for both ovaries (Table 1).

**Table 1:** Demographic and reproductive characteristics of PCOS women

Variables	Groups	n (%)
Age group (years) 28.24 (4.84)*	15–25	16 (31.4)
	26–35	32 (62.7)
	36–45	3 (5.9)
Marriage duration (years) 4.62 (3.58)*	< 1	6 (11.8)
	1–5	32 (62.7)
	> 5	13 (25.5)
Fertility status	Primary	40 (78.4)
	Secondary	11 (21.6)
Past treatment	None	10 (19.6)
	Ovarian induction	31 (60.8)
	Multiple	10 (19.6)
Weight (kg) 75.22 (13.71)*	55–75	31 (60.8)
	76–95	16 (31.4)
	96–115	4 (7.8)
Hirsutism (MFG score)	None (≤ 7)	5 (9.8)
	Mild (8–15)	22 (43.1)
	Moderate (16–25)	18 (35.3)
	Severe (26–34)	6 (11.8)
Menstrual cycle length 39.29 (25.35) (days)	Normal	18 (35.3)
	Oligomenorrhea	18 (35.3)
	Amenorrhea	15 (29.4)
Left ovary volume	Without PCOM (Vol < 10 cm^3^)	5 (9.8)
	PCOM (Vol > 10 cm^3^)	46 (90.2)
Right ovary volume	Without PCOM (Vol < 10 cm^3^)	8 (15.7)
	PCOM (Vol > 10 cm^3^)	43 (84.3)

^*^Mean (SD)

PCOS women (70.6%) showed high AMH (> 3.90 ng/ml) levels when measured for reproductive hormonal levels in the serum. FSH (< 9 mlU/ml), LH (< 12.5 IU/L), prolactin (< 29 ng/ml), and TSH (< 4.0 mmIU/L) were found to be normal among the majority of the participants (Table 2).

**Table 2:** Reproductive hormone levels among PCOS women

Variable	Groups	n (%)	Mean (SD)
Follicle stimulating hormone (FSH)	Normal (≤ 9 mlU/ml)	49 (96.1)	5.63 (1.79)
	High (≥ 9 mlU/ml)	7 (15.7)	
Luteinizing hormone (LH)	Normal (≤ 12.5 IU/L)	43 (84.3)	8.58 (5.45)
	High (≥ 12.5 IU/L)	8 (15.7)	
Prolactin	Normal (≤ 29 ng/ml)	45 (88.2)	16.68 (13.71)
	High (≥ 29 ng/ml)	6 (11.8)	
Anti-Müllerian hormone (AMH)	Normal (≤ 3.9 ng/ml)	15 (29.4)	7.23 (4.67)
	High (≥ 3.9 ng/ml)	36 (70.6)	
Thyroid stimulating hormone (TSH)	Normal (≤ 4.0 mmIU/L)	50 (98.0)	1.94 (0.82)
	High (≥ 4.0 mmIU/L)	1 (2.0)	

The independent t test showed a significant mean difference (p < 0.05) for AMH and LH reproductive hormones when compared between normal and oligo-/amenorrhea menstrual patterns (Table 3). Mean group comparison between PCOS women with normal and high AMH levels showed a significant relationship (p < 0.05) between left ovary volume and LH hormone. PCOM among women can be used as an important clinical feature to diagnose PCOS. Based on previous studies’ evidence, a threshold of 3.9 ng/mL was considered normal in the present study to make a group comparison of PCOS women with higher AMH levels (Table 4). PCOS women (70.6%) showed a higher AMH level (≥ 3.9 ng/ml).

**Table 3:** Comparison of reproductive hormone levels between the normal and oligo-/amenorrhea groups

Reproductive hormone	PCOS women menstrual cycle length		Mean difference^a^ (95% CI)
	Normal (Mean ± SD)	Oligo-/Amenorrhea (Mean ± SD)	
Follicle Stimulating Hormone (FSH)	5.52 ± 1.78	5.69 ± 1.82	0.75 (− 1.23, 0.89)
Luteinizing Hormone (LH)	6.67 ± 2.46	9.62 ± 6.33	0.02* (− 5.46, − 0.44)
Prolactin	19.85 ± 20.40	14.96 ± 8.01	0.34 (− 5.55, 15.34)
Anti-Müllerian hormone (AMH)	5.34 ± 3.19	8.27 ± 5.05	0.03* (− 5.57, − 0.28)
Thyroid Stimulating Hormone (TSH)	1.97 ± 0.92	1.94 ± 0.77	0.90 (− 0.45, 0.52)

^a^Independent t test, *p value < 0.05

**Table 4:** Comparison of reproductive characteristics between normal and high AMH levels among PCOS women

Reproductive outcome measure	Anti-Müllerian hormone level among PCOS women		Mean difference ^a^ (95% CI)
	Normal (less than 3.9 ng/ml) (Mean ± SD)	High (greater than 3.9 ng/ml) (Mean ± SD)	
Age	29.67 ± 5.32	27.93 ± 4.74	0.38 (− 1.83, 5.31)
Average menstrual cycle days	30.33 ± 15.51	41.21 ± 26.33	0.25 (− 29.52, 7.76)
Left ovary volume	18.40 ± 6.36	14.91 ± 4.30	0.05* (0.02, 6.95)
Right ovary volume	13.81 ± 5.72	14.36 ± 3.61	0.71 (− 3.53, 2.42)
Follicle stimulating hormone (FSH)	5.82 ± 1.96	5.58 ± 1.77	0.73 (− 1.10, 1.56)
Luteinizing hormone (LH)	5.63 ± 1.43	9.21 ± 5.78	0.01* (− 7.52, 0.35)
Prolactin	15.30 ± 11.05	16.98 ± 14.31	0.73 (− 11.88, 8.54)
Thyroid stimulating hormone (TSH)	2.26 ± 0.82	1.87 ± 0.80	0.20 (− 0.21, 0.98)

^a^Independent t test, *p value < 0.05

**Fig. 1:**
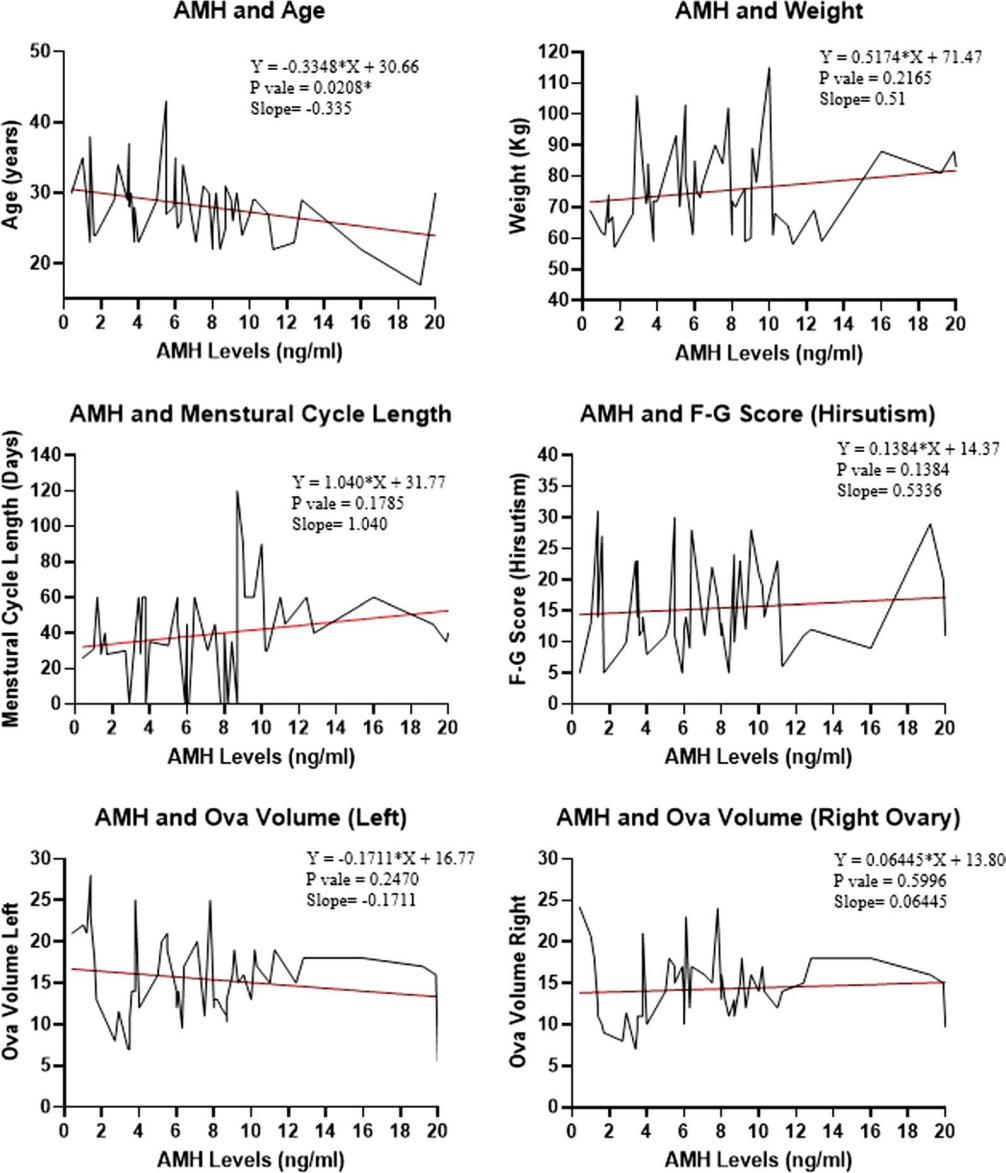
Linear regression model for AMH levels and PCOS characteristics

**Fig. 2:**
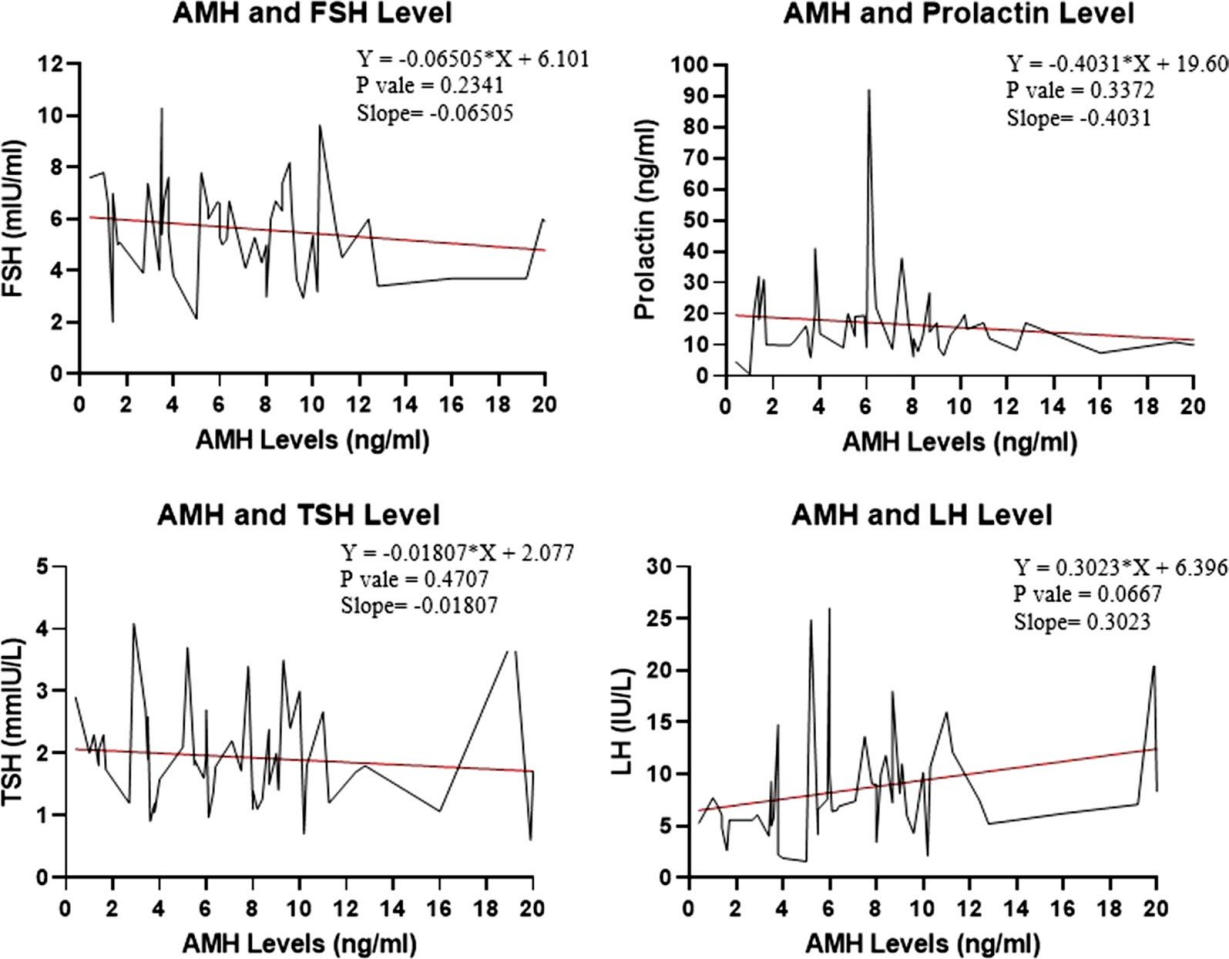
Linear regression model for AMH and reproductive hormonal levels among PCOS women

A linear regression model was used to predict the pattern of AMH with various reproductive characteristics and serum levels of hormones, as shown in Figs. 1 and 2. The slope of the regression line showed a significant decline in serum AMH levels with an increase in age. AMH levels tend to increase with weight, menstrual abnormalities, and hirsutism. LH was the only reproductive hormone that increased with the elevation of serum AMH levels among PCOS women.

## Discussion

Our study found that PCOS women had an increased menstrual cycle length with the elevation in serum AMH levels and had a greater risk of menstrual disturbance. The increase in menstrual cycle length with AMH elevation was concurrent with previous studies [26]. Serum AMH is highly correlated with the antral follicle count, which acts as a biomarker for an ovarian response [27]. Serum AMH levels can be a predictive tool for oligo-/amenorrhea with higher per antral secretion among PCOS women.

The results of this study found that the risk of oligo-/amenorrhea increases with higher serum AMH levels, and it was difficult to define a single threshold value. A trend towards an increase in menstrual cycle length was obvious when compared with the increase in AMH level. A threshold AMH level between 3.8 and 5 ng/mL has been reported previously to diagnose PCOS [18]. The serum AMH concentration in PCOS patients has also been reported to be high in previous studies when compared with BMI and age-matched controls [28]. As a quantitative laboratory parameter, serum AMH can be used for the assessment of ovarian reserve by taking a blood sample on any day of the cycle [29].

Serum AHM levels also showed a significant relationship (p = 0.01) with LH and tended to increase LH secretion with higher AMH levels. The predominant secretion of LH among PCOS women also had a significant relationship (p = 0.02) with menstrual disturbance. Similar findings were reported in another study suggesting that GnRH made LH predominance in the follicular phase commensurate with higher AMH levels and menstrual disturbance [30].

Abnormalities of increased LH secretion have been reported among PCOS women > 40 years as a result of accelerated GnRH/LH pulsatility. The FSH levels were found to remain normal, resulting in an increased LH/FSH ratio during the follicular phase of the menstrual cycle among PCOS women [31]. The LH/FSH ratio depends on the assay used to measure these gonadotropins, making it difficult to define a cut-off value and its application in clinical settings [32]. For these reasons, gonadotropin measurements are not evident for PCOS diagnosis.

Hyperandrogenism is one of the Rotterdam criteria to diagnose PCOS and is prominent among PCOS women with increased BMI. PCOS women with high follicle counts and increased BMI levels were prone to biochemical hyperandrogenism [30]. Clinical features of hyperandrogenism have frequently been seen among PCOS women, including hirsutism, acne, and androgenic alopecia [32]. Hirsutism is one of the auspicious features of hyperandrogenism and was found to be prevalent among our study participants.

A positive correlation between serum AMH levels and androgen has previously been reported. Hyperandrogenism has been reported as an intrinsic defect of thecal cells in PCOS women and is positively associated with testosterone levels and ovarian volume [33]. The clinical symptoms of hirsutism and menstrual cycle abnormalities were also reported to be high in our study. A trend of increase in body weight and hirsutism was observed when compared with elevation in serum AMH levels.

In our data, PCOM in both ovaries was present in the majority of participants. The PCOS women had PCOM, hyperandrogenism, and oligo-/amenorrhea along with increased serum AMH levels. The findings of this study suggest that AMH can be used as a biochemical marker to reliably identify PCOS based on the clinical features defined by the Rotterdam criteria. The combination of AMH with the Rotterdam criteria can better identify PCOS. Teede et al. [34] summarized the potential challenges to using serum AMH levels to diagnose PCOS, which needs to be incorporated for the effective use of this diagnostic tool.

PCOS remains a syndrome, and as such, no single diagnostic criteria are sufficient for clinical diagnosis. Environmental factors have also been reported to play an essential role in PCOS pathogenesis [35], which is beyond the scope of our research. Similarly, genetics also play a momentous role in the origin of this disease [36]. The phenotypic presentation of PCOS women can be altered by baseline variables such as age, BMI, physical activity level, and dietary patterns, which limits the findings of this study. The potential limitation of our data includes the small sample size and purposive sampling of women visiting the outdoor fertility center. This study did not compare the serum AMH levels with age- and BMI-matched non-PCOS women to estimate the normal ranges and trends. Considering the cross-sectional nature of the study, the participants were not observed over a longer period with follow-up biochemical analysis of serum AMH and other reproductive hormones to make an intrarater comparison.

The strengths of this study include that specialist gynaecologists screened the newly diagnosed PCOS women and had the expertise of accurate assessment of ovarian morphology on ultrasound along with other PCOS features, resulting in reduced inter- and intraobserver bias. This study addresses an unattended research area in Pakistan and highlights the importance of AMH in the accurate diagnosis of PCOS. Future research with broader baseline variables, comparison with healthy controls, and studying PCOS women cohorts for a longer period could yield further interesting findings in utilizing the serum AMH level as a diagnostic tool for PCOS.

This study has the strength of being novel in Pakistan settings to introduce serum AMH level as a diagnostic biomarker in Pakistan. This study used the Rotterdam criteria for PCOS identification and tried to use serum AMH levels as an auxiliary method for the identification of ovarian dysfunction and polycystic ovarian morphology. This cross-sectional study has the limitation of not analysing the serum AMH level of PCOS women with healthy controls. During the planning stage, it was observed that the healthy controls refused to undergo a transvaginal ultrasonogram for the possible social and religious stigmas attached to this investigational method. To overcome this limitation an intragroup comparison of PCOS women with a serum AMH level (< 3.9 ng/mL vs. > 3.9 ng/mL) was made to compare the Rotterdam criteria.

Another limitation is that PCOS women were measured for their bodyweight only. That data were collected from the outdoor fertility clinic with limited female staff, and the unavailability of a standardized apparatus to measure the anthropometric measurements restrained the researcher from collecting the additional parameters to calculate BMI and waist/hip ratio.

Another case–control study design was conducted in a similar research area with the possible recruitment of healthy controls for biochemical and lifestyle comparisons with PCOS women. Anthropometric measurements, including waist/hip ratio, were measured by the lady health worker using standardized measuring apparatuses.

## Conclusions

An accurate and appropriate PCOS diagnosis is essential, as it has long-term implications on women’s health. An elevated serum AMH level can be used as a strong predictor to reflect the certainty of PCOS diagnosis among women of reproductive age when study concurrently with the other Rotterdam criteria. The use of serum AMH is probably easier to perform than the use of an ultrasonogram to estimate ovarian reserves.

## Data Availability

The dataset generated and analysed during the current study is not publicly available because participants' consent only referred to a publication of aggregated data. However, the dataset can be made available from the corresponding author on reasonable request.

## Abbreviations

AMH: Anti-Müllerian hormone
ELISA: Enzyme-linked immunosorbent assay
FSH: Follicle-stimulating hormone
GnRH: Gonadotropin-releasing hormone
LH: Luteinizing hormone
M: Mean
MFG: Modified Ferriman-Gallwey
PCOM: Polycystic ovarian morphology
PCOS: Polycystic ovarian syndrome
SD: Standard deviation
TSH: Thyroid-stimulating hormone
